# Incidence and associations of hospital delirium diagnoses in 85,979 people with severe mental illness: A data linkage study

**DOI:** 10.1111/acps.13480

**Published:** 2022-08-05

**Authors:** Yehudit Bauernfreund, Naomi Launders, Graziella Favarato, Joseph F. Hayes, David Osborn, Elizabeth L. Sampson

**Affiliations:** ^1^ Division of Psychiatry University College London London UK; ^2^ Camden & Islington NHS Foundation Trust London UK; ^3^ Department of Psychological Medicine East London NHS Foundation Trust, Royal London Hospital London UK

**Keywords:** bipolar disorder, databases, delirium, psychotic disorders, schizophrenia

## Abstract

**Introduction:**

Delirium is an acute neuro‐psychiatric disturbance precipitated by a range of physical stressors, with high morbidity and mortality. Little is known about its relationship with severe mental illness (SMI).

**Methods:**

We conducted a retrospective cohort study using linked data analyses of the UK Clinical Practice Research Datalink (CPRD) and Hospital Episodes Statistics (HES) databases. We ascertained yearly hospital delirium incidence from 2000 to 2017 and used logistic regression to identify associations with delirium diagnosis in a population with SMI.

**Results:**

The cohort included 249,047 people with SMI with median follow‐up time in CPRD of 6.4 years. A total of 85,979 patients were eligible for linkage to HES. Delirium incidence increased from 0.04 (95% CI 0.02–0.07) delirium associated admissions per 100 person‐years in 2000 to 1.05 (95% CI 0.93–1.17) per 100 person‐years in 2017, increasing most notably from 2010 onwards. Delirium was associated with older age at study entry (OR 1.05 per year, 95% CI 1.05–1.06), SMI diagnosis of bipolar affective disorder (OR 1.66, 95% CI 1.44–1.93) or other psychosis (OR 1.56, 95% CI 1.35–1.80) relative to schizophrenia, and more physical comorbidities (OR 1.08 per additional comorbidity of the Charlson Comorbidity Index, 95% CI 1.02–1.14). Patients with delirium received more antipsychotic medication during follow‐up (1–2 antipsychotics OR 1.65, 95% CI 1.44–1.90; >2 antipsychotics OR 2.49, 95% CI 2.12–2.92).

**Conclusions:**

The incidence of recorded delirium diagnoses in people with SMI has increased in recent years. Older people prescribed more antipsychotics and with more comorbidities have a higher incidence. Linked electronic health records are feasible for exploring hospital diagnoses such as delirium in SMI.

**Significant Outcomes**

- Incidence of recorded in‐hospital delirium diagnoses in people with severe mental illness (SMI) has increased in recent years, from 0.04 (95% CI 0.02–0.07) delirium associated admissions per 100 person‐years in 2000 to 1.05 (95% CI 0.93–1.17) per 100 person‐years in 2017, with the increase most notable from 2010 onwards.
- Key associations with delirium diagnosis in people with SMI are older age, SMI diagnosis of bipolar affective disorder (BPAD) or other psychosis, more antipsychotic prescriptions during follow‐up, and more physical comorbidities.
- Linked primary and secondary electronic health records are suitable for exploring hospital diagnoses such as delirium in SMI, and allow for identification of further exposure variables compared with using hospital data only.

**Limitations**

- Delirium incidence was measured using coding in hospital data which is likely to under‐estimate incidence compared with prospective screening measures.
- Delirium incidence was ascertained from hospital data only, and not from community data.

## 1 INTRODUCTION

Delirium is an acute clinical syndrome causing cognitive and neuro‐psychiatric disturbance, associated with adverse outcomes and high mortality.^1^ Several underlying health conditions have been well‐characterised as pre‐disposing factors, including dementia and frailty.^2^, ^3^ However, the relationship between pre‐existing mental illness and delirium is less well‐understood. Several studies have demonstrated an association between depression and delirium, with depression increasing the risk of delirium by up to nine times.^4^ Severe mental illness (SMI) is defined by the United Kingdom quality and outcomes framework (QoF; a framework for monitoring the management of major public health concerns in primary care)^5^ as including schizophrenia, bipolar affective disorder and other non‐organic psychoses. Despite an established association with depression, little is known about the relationship between delirium and other types of mental illness. People with SMI are disproportionately affected by physical health problems, are high users of secondary care,^6^ and understanding and improving health outcomes for this group is a public health priority.^7^

People with SMI have high rates of physical comorbidity, exposure to long‐term psychotropic medications, reduced cognitive reserve and markers of frailty^8^, ^9^, ^10^, ^11^, ^12^; all factors known to be associated with delirium.^2^ However, studies describing the occurrence of delirium in psychiatric populations are scarce and derived from whole psychiatric populations, rather than SMI specifically.^13^, ^14^, ^15^, ^16^ A further specific concern in SMI is the overlap in symptomatology with delirium, which may lead to under‐diagnosis of delirium because of diagnostic overshadowing.^17^, ^18^ Under‐recognition of delirium is associated with increased mortality.^19^ We urgently need a better understanding of the frequency and determinants of delirium in this population to address diagnostic delays, direct prevention and screening strategies and to improve individual and health system outcomes. In addition, because of the overlapping symptomatology between SMI and delirium, we wanted to assess the temporal relationship between these conditions in patients with both diagnoses recorded, in order to clarify whether SMI precedes delirium, whether it can follow delirium, and explore possible misdiagnosis in this group.

The use of electronic health records (EHRs) for population health research has increased greatly in the last decade.^20^ EHRs capture data on a wide range of epidemiological variables for large numbers of real‐world patients, and linked EHRs can explore variables included in different sources including primary and secondary (hospital) care. They are of particular value in researching populations with stigmatised disorders such as SMI, when recruitment and follow‐up may be challenging.^20^ Studies of delirium incidence and risk factors are typically conducted in small hospital‐based cohorts,^21^ and few existing studies have used large scale national databases to study this.^16^, ^22^, ^23^, ^24^ Linked EHRs offer an opportunity to improve understanding of delirium occurrence in SMI.

## 2 STUDY AIMS

Our primary aims were to:

1. Describe the yearly incidence of delirium diagnoses in a community‐based cohort of patients with SMI derived from Clinical Practice Research Datalink (CPRD), using linkage to Hospital Episode Statistics (HES) data.
2. Establish factors associated with delirium in a population with SMI.
3. Establish the temporal relationship between SMI diagnoses and delirium diagnoses within linked EHRs.

Our secondary methodological aims were to:

4 Evaluate the utility of linked primary and secondary EHRs for investigating delirium in SMI.
5 Explore differences in exposure variables derived from different primary care databases (CPRD GOLD and CPRD Aurum).

## 3 MATERIALS AND METHODS

### 3.1 Study design

Cohort study using anonymised linked EHRs, reported according to the STROBE guidelines for cohort studies.^25^

### 3.2 Data sources

The CPRD is a primary‐care database of anonymised patient records encompassing 60 million patients from 2000 UK practices.^26^ It comprises two databases, GOLD and Aurum. GOLD contains data from the Vision electronic record system and collects UK wide data, whilst Aurum contains data from the increasingly used EMIS system, currently from England only.^27^ Few other studies describe and compare data from both GOLD and Aurum databases.^28^, ^29^, ^30^, ^31^, ^32^ As more UK primary care practices move over to the EMIS system, more studies comparing Aurum and GOLD are needed. A proportion of CPRD data can be linked to other databases, including Hospital Episodes Statistics Admitted Patient Care (HES‐APC). HES‐APC contains data on all NHS admissions in England, including both general hospital and psychiatric admissions.^33^ It includes data on separate admissions, consultant episodes within an admission, and unique diagnoses within episodes. It contains duplicate records of unique diagnostic episodes and requires a systematic cleaning approach (see Appendix S2). We performed data linkage on a diagnosis level to retain episodes of delirium that were not the primary diagnosis for a general hospital or psychiatric admission.

### 3.3 Study population

#### 3.3.1 CPRD SMI cohort

We included all patients registered with a CPRD general practice for at least 1 year between 01 January 2000 and 31 December 2018, who had received a diagnosis of SMI prior to or at any time during follow‐up. We identified patients with SMI using medical Read codes for schizophrenia, bipolar affective disorder or other non‐organic psychosis (code lists available on request). For the main SMI cohort, we excluded patients who received a diagnosis of SMI after exiting the cohort and those who left the cohort aged ≥100 years or ≤16 years. We followed patients from 01 January 2000 or registration date (latest) until transfer out of practice date, death or 31 December 2018 (earliest).

#### 3.3.2 CPRD‐HES‐APC linkage

We identified patients in the CPRD SMI cohort, eligible for linkage to HES‐APC for records 01 January 2000–31 December 2017. We analysed admissions occurring at ≥16 years to explore the relationship between SMI and delirium in adults only. We compared characteristics between those eligible for HES linkage to those not eligible for linkage to assess for linkage bias.

### 3.4 Outcomes

Our primary outcome was yearly incidence rate of in‐hospital delirium within our linked CPRD‐HES cohort. We identified delirium in HES‐APC using ICD‐10 codes. As recent expert consensus includes the pathological process of acute encephalopathy within the clinical syndrome of delirium,^34^ we included both delirium codes (F05) and acute encephalopathy codes (G04, G92, G93; for full list, see Appendix 3, Table S4). We included all general hospital and psychiatric admissions that included an episode of delirium. As one episode of delirium may be recorded multiple times during an admission, we report incidence of delirium associated admissions. We chose to include delirium episodes that occurred before SMI diagnosis (i.e., SMI diagnosis could be recorded up to 31 December 2018) to allow for prodromal illness and reporting delay in SMI diagnoses^35^, ^36^ and in order to explore the temporal relationship between these conditions within electronic healthcare records.

### 3.5 Covariates

We extracted data on demographic variables including age at study entry, sex, and ethnicity from CPRD. We categorised ethnicity as Asian, Black, White, Mixed or Other, and missing ethnicity re‐classified as ‘White’, in line with previously validated methods.^37^ We extracted data on clinical variables using Read codes in CPRD. We categorised SMI diagnosis as schizophrenia (including schizo‐affective disorders), Bipolar Affective Disorder or other non‐organic psychotic illness; number of different antipsychotics prescribed at any point during follow‐up as none, 1–2 antipsychotics, or >2± depot, and physical comorbidities at cohort entry using the Charlson Comorbidity Index.^38^ We extracted hospitalisations per year of follow‐up from HES. For delirium associated admissions, we extracted data from HES on age at admission, primary diagnosis for admissions and duration of admissions.

### 3.6 Statistical analysis

We calculated incidence rate as delirium associated hospital admissions per 100 person‐years at risk for each year of cohort follow‐up. Patients eligible for linkage contributed to the person‐years at risk for the duration they remained in CPRD follow‐up. We explored the association between our covariates and odds of in‐hospital delirium diagnosis using logistic regression analyses to calculate odds ratios (ORs) with 95% confidence intervals (CIs). We calculated unadjusted ORs and multivariate adjusted ORs to control for the possible confounding effects of age, sex, ethnicity, SMI diagnosis and antipsychotics during follow‐up. We did not adjust for physical comorbidities in our multivariate analysis as these are widely considered to lie on the causal pathway. This is because of the widely established finding of SMI as a risk factor for physical multi‐morbidity and frailty,^8^, ^39^, ^40^, ^41^ and physical multi‐morbidity and frailty as known predisposing factors for delirium.^2^, ^42^, ^43^ We conducted a sensitivity analysis using the subset with any hospitalisation during follow‐up to identify associations specifically for delirium, rather than any hospital admission. We used STATA 16 for data linkage, cleaning and analysis.

### 3.7 Ethics

The study was approved by the Independent Scientific Advisory Committee of CPRD (protocol no. 18_288), waiving informed consent because data are anonymised for research purposes.

## 4 RESULTS

### 4.1 Participants

**FIGURE 1:**
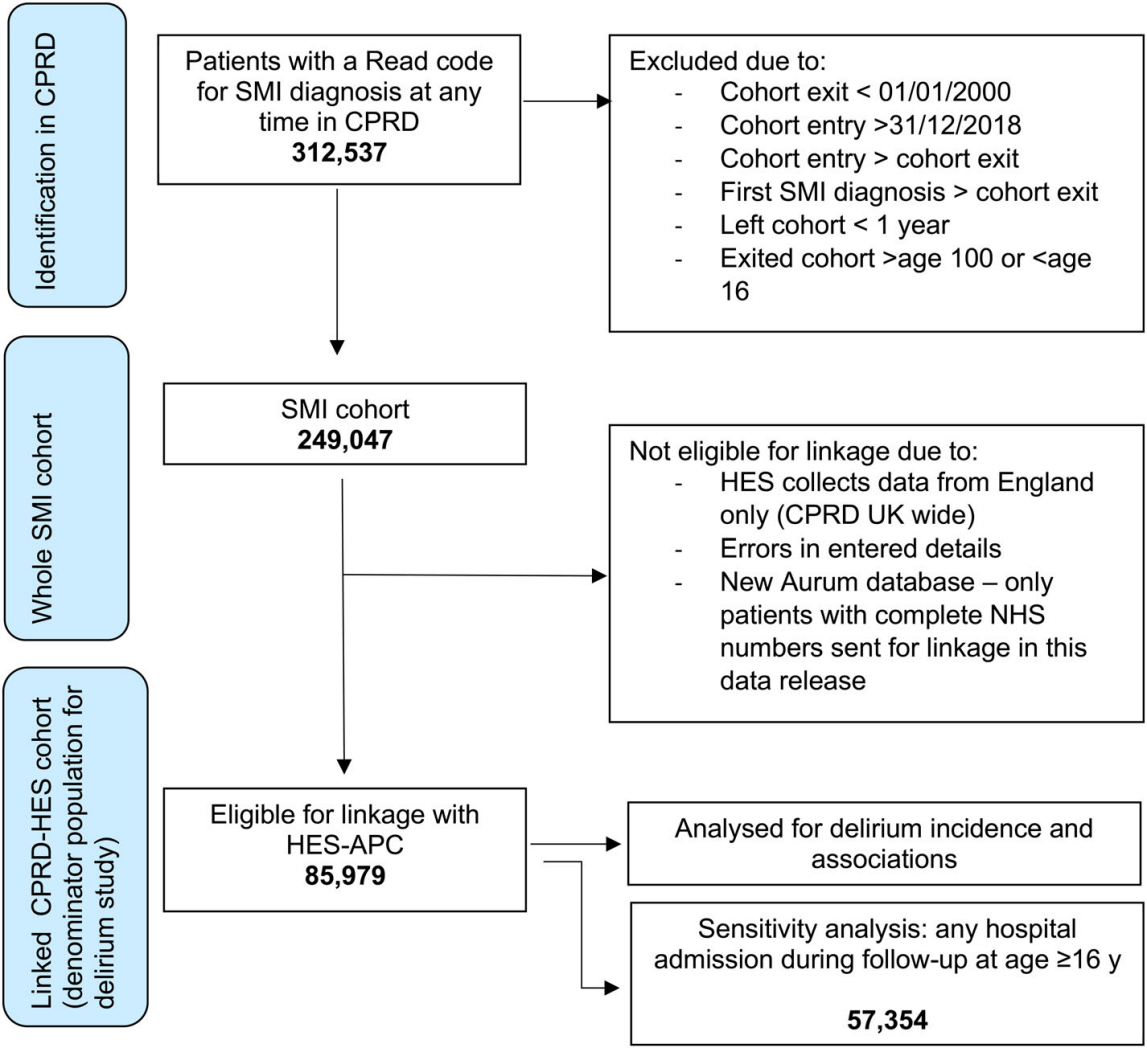
STROBE flow diagram of participants

We identified 312,537 patients with a Read code for SMI diagnosis at any time in CPRD. 249,047 (79.7%) of these patients met the study inclusion criteria; 87,255 in GOLD and 161,792 in Aurum. Aurum patients tended to be born and enter and exit the cohort at a later period than GOLD patients, and were more likely to have ethnicity recorded (see Appendix S1 for full comparison). Of these 249,047 patients, 85,979 (34.5%) were eligible for data linkage with HES‐APC (40,738 (46.7%) in GOLD and 45,241 (28.0%) in Aurum). Patients eligible for linkage did not differ significantly from those not eligible for linkage (see Appendix 1, Table S3). During follow‐up, 1337 (1.6%) patients eligible for HES linkage had ≥1 admission involving delirium (Figure 1). 57,354 (66.6%) patients had any admission to hospital and were used for sensitivity analysis.

### 4.2 Cohort characteristics

Characteristics of patients eligible for linkage are presented in Table 1 (for characteristics of whole SMI cohort, see Appendix S1). Median age of participants at study entry was 42 years and 48.5% were female. 86.5% were of White ethnicity. 32.1% had schizophrenia, 32.4% had bipolar affective disorder and 35.5% had other non‐organic psychosis. During follow‐up, 32.7% were prescribed no antipsychotics, 49.4% were prescribed 1–2 antipsychotics and 19.9% were prescribed >2± a depot antipsychotic. The number of Charlson Comorbidity Index conditions at cohort entry ranged from 0 to 8 with median 0 (IQR 0–1). Hospitalisations per year of follow‐up ranged from 0 to 4.20 with median 0.22 (IQR 0–0.61). 13.9% died during follow‐up at median age 76 (IQR 62–85).

**TABLE 1:** Demographic and clinical characteristics of patients within the linked cohort

*N*	CPRD‐HES linked cohort 85,979	
	*N*/median	% (95% CI)/IQR
Source		
CPRD GOLD	40,738	47.4 (47.0–47.4)
CPRD Aurum	45,241	52.6 (52.3–53.0)
Age at study entry (years)	42	30–58
Follow‐up time (years)	6.2	2.9–12.4
Gender		
Female	41,693	48.5 (48.2–48.8)
Male	44,286	51.5 (51.2–51.8)
Ethnicity		
Asian	3870	4.5 (4.4–4.6)
Black	4676	5.4 (5.3–5.6)
White	74,358	86.5 (86.3–86.7)
Mixed	1088	1.3 (1.2–1.3)
Other	1987	2.3 (2.2–2.4)
SMI diagnosis		
BPAD	27,848	32.4 (32.1–32.7)
Schizophrenia	27,570	32.1 (31.8–32.4)
Other psychosis	30,561	35.5 (35.2–35.9)
No. of antipsychotics		
None	28,111	32.7 (32.4–33.0)
1–2 APs	42,456	49.4 (49.0–49.7)
>2 Aps ± depot	15,412	17.9 (17.7–18.2)
Charlson comorbidity score	0	0–1
No. of hospitalisations per year of FU	0.22	0–0.61
Died during FU	11,917	13.9 (13.6–14.1)
Age at death (years)	76	62–85

*Note*: 95% confidence intervals displayed around proportions.

Abbreviations: Aps, antipsychotics; BPAD, bipolar affective disorder; CI, confidence interval; FU, follow‐up; IQR, interquartile range.

### 4.3 Incidence of delirium

**FIGURE 2:**
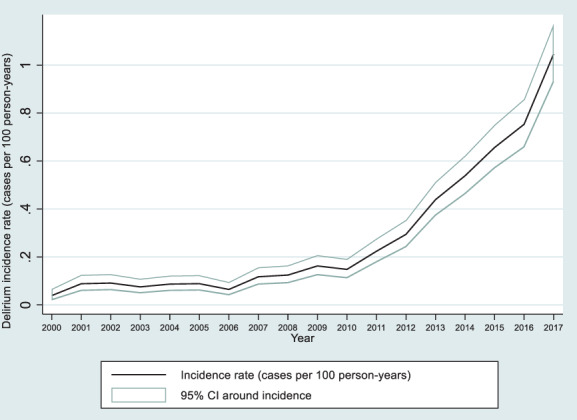
Yearly incidence rates (cases per 100 person‐years for each year of follow‐up) of delirium within our linked cohort, with 95% confidence intervals displayed. CI, confidence Interval

There were 1689 delirium associated admissions in total during follow‐up, with 1337 of 85,979 unique patients (1.6%) experiencing ≥1 delirium associated admission. 1075 (80.4%) had a single delirium associated admission, and 262 (19.6%) had multiple delirium associated admissions. Delirium incidence increased from 0.04 (95% CI 0.02–0.06) cases per 100 person‐years in 2000 to 1.05 (0.93–1.17) cases per 100 person‐years in 2017 (see Appendix 3, Table S5). Incidence rate increased steeply from 2010 onwards (Figure 2). The most common primary diagnoses for the 1689 delirium associated admissions were delirium itself (24.9%), urinary tract infection (11.5%) and lower respiratory tract infections (9.2%). Median age at time of delirium was 76 (IQR 65–84). Median duration of delirium associated admissions was 12 days (IQR 5–26.5).

### 4.4 Factors associated with delirium‐related admissions

Odds of receiving a diagnosis of in‐hospital delirium increased for every year increase in age at study entry (OR 1.05, 95% CI 1.05–1.06), and this remained significant (*p* < 0.001) when adjusting for age, sex, ethnicity, SMI diagnosis and antipsychotics during follow‐up (fully adjusted model). In the unadjusted logistic regression, female sex was significantly associated with higher odds of delirium diagnosis (OR 1.66, 95% CI 1.57–1.97, *p* < 0.001), and people of White ethnicity had higher odds compared with those of Asian (OR 0.59, 95% CI 0.42–0.82, *p* = 0.002), Black (OR 0.57, 95% CI 0.42–0.78, *p* < 0.001) and Mixed (OR 0.49, 0.25–0.94, *p* = 0.032) ethnicities. However, these associations were not observed in the age and sex adjusted and fully adjusted models. An SMI diagnosis of schizophrenia was associated with lower odds of in‐hospital delirium diagnosis compared with BPAD (OR 1.66, CI 1.44–1.93) and other psychosis (OR 1.56, CI 1.35–1.80), and this remained significant (*p* < 0.001) in the fully adjusted model. Prescription of more antipsychotics during follow‐up was associated with increased odds of in‐hospital delirium (1–2 APs; OR 1.65 CI 1.44–1.90, >2 APs± depot OR 2.49 CI 2.12–2.92), and this remained significant (*p* < 0.001) in the fully adjusted model. Odds of delirium increased by 1.08 (1.02–1.14) for every additional CCI condition (Table 2).

**TABLE 2:** Odds ratios for receiving an in‐hospital delirium code within the linked CPRD‐HES cohort (*n* = 85,979)

	Unadjusted			Age and sex adjusted			Fully adjusted		
Exposure	Odds ratio	95% CI	*p*‐Value	OR	95% CI	*p*‐Value	OR	95% CI	*p*‐Value
Age at study entry	1.05	1.05–1.06	<0.001	1.05	1.05–1.05	<0.001	1.05	1.05–1.06	<0.001
Gender									
Male	Ref			Ref			Ref		
Female	1.76	1.57–1.97	<0.001	1.08	0.96–1.22	0.193	1.00	0.89–1.12	0.952
Ethnicity									
White	Ref			Ref			Ref		
Asian	0.59	0.42–0.82	0.002	0.97	0.69–1.35	0.839	0.98	0.70–1.38	0.925
Black	0.57	0.42–0.78	<0.001	0.96	0.70–1.32	0.821	1.04	0.76–1.44	0.786
Mixed	0.49	0.25–0.94	0.032	1.01	0.52–1.95	1.953	1.07	0.55–2.09	0.831
Other	1.11	0.79–1.57	0.542	1.23	0.86–1.74	0.253	1.23	0.87–1.74	0.247
SMI diagnosis									
Schizo‐phrenia	Ref			Ref			Ref		
BPAD	1.44	1.25–1.67		1.48	1.28–1.72	<0.001	1.66	1.44–1.93	<0.001
Other psychosis	1.62	1.41–1.87	<0.001 < 0.001	1.45	1.26–1.67	<0.001	1.56	1.35–1.80	<0.001
Antipsychotics during follow‐up									
None	Ref			Ref			Ref		
1–2 APs	1.61	1.40–1.85	<0.001	1.62	1.41–1.86	<0.001	1.65	1.44–1.90	<0.001
>2 APs ± depot	2.15	1.83–2.52	<0.001	2.28	1.95–2.68	<0.001	2.49	2.12–2.92	<0.001
Physical comorbidities	1.58	1.50–1.66	<0.001	1.08	1.02–1.14	0.013	*		

Abbreviations: Aps, antipsychotics; BPAD, bipolar affective disorder; CCI, Charlson Comorbidity Index; CI, confidence interval; Ref = reference.

^a^ Physical comorbidities were not included in the fully adjusted model as confounding factors as they are likely to lie on the causal pathway.

In the sensitivity analysis, limiting the cohort to those with a hospital admission during follow‐up, to explore whether these exposures are associated with delirium associated hospital admissions specifically, did not alter the results (see Appendix 4, Table S6).

### 4.5 Temporal relationship between SMI and delirium associated admissions

The majority of patients (81.1%) received their SMI diagnosis before experiencing any delirium‐associated admission. The 18.9% of patients who experienced a delirium associated admission prior to SMI diagnosis did not differ significantly in demographic characteristics, however, were more likely to have SMI diagnosis of other psychosis and less likely to have a diagnosis of schizophrenia. They were also more likely to be prescribed 1–2 antipsychotics during follow‐up and less likely to be prescribed >2 antipsychotics ± depot during follow‐up, and more likely to die during follow‐up (Table 3).

**TABLE 3:** Comparison of group who received SMI diagnosis before delirium diagnosis to those who received SMI diagnosis after delirium diagnosis. 95% confidence intervals displayed around proportions

*N*	SMI before delirium dx 1084 (81.1%)		SMI after delirium dx 253 (18.9%)	
	*N*/median	% (95% CI)/IQR	*N*/median	% (95% CI)/IQR
Source				
GOLD	444	41.0 (38.1–44.0)	96	37.9 (31.9–44.2)
Aurum	640	59.0 (56.0–62.0)	157	62.1 (55.8–68.1)
Age at study entry (years)	66	55–75	63	52–73
Follow‐up time (years)	11.3	5.1–16.2	13.0	7.2–17.6
Gender				
Female	667	61.5 (58.6–64.4)	164	64.8 (58.6–70.7)
Male	417	38.5 (35.6–41.1)	89	35.2 (29.3–41.4)
Ethnicity				
Asian	32	3.0 (2.0–4.1)	6	2.4 (0.9–5.1)
Black	34	3.1 (2.2–4.4)	10	4.0 (1.9–7.1)
White	984	90.8 (88.9–92.4)	229	90.5 (86.2–93.8)
Mixed	7	0.6 (0.3–1.3)	0	0.0 (0.0–0.0)
Other	27	2.5 (1.6–3.6)	8	3.2 (1.4–6.4)
SMI diagnosis				
BPAD	386	35.6 (32.8–38.5)	72	28.5 (23.0–34.4)
Schizophrenia	292	26.9 (24.3–29.7)	24	9.5 (6.7–13.8)
Other psychosis	406	37.5 (34.6–40.4)	157	62.1 (55.8–68.1)
No. of antipsychotics				
None	243	22.4 (20.0–25.0)	49	19.4 (14.7–24.8)
1–2 APs	540	49.8 (46.8–52.8)	165	65.2 (59.0–71.1)
>2 Aps ± depot	301	27.8 (25.1–30.5)	39	15.4 (11.1–20.5)
≥1 comorbidity on CCI				
	513	47.3 (44.3–50.3)	116	45.9 (39.6–52.2)
No. of hospitalisations per year of FU	0.89	0.51–1.50	0.82	0.43–1.34
Died during FU	442	40.8 (37.8–43.8)	72	28.5 (23.0–34.4)
Age at death (years)	81	72–86	82.5	73–88.5

Abbreviations: Aps, antipsychotics; BPAD, bipolar affective disorder; CI, confidence interval; FU, follow‐up; IQR, interquartile range.

## 5 DISCUSSION

In this large retrospective cohort covering 18 years of follow‐up, we report the incidence of delirium in people with SMI. Incidence increased from 0.04 cases per 100 person‐years in 2000 to 1.05 cases per 100 person‐years in 2017, with a steep increase from 2010 onwards. Previous studies reporting delirium occurrence in psychiatric populations are focused on whole psychiatric cohorts, including patients with dementia, depression, anxiety and substance misuse, rather than SMI specifically. The proportion of psychiatric inpatients who develop delirium varies from 1.4% to 14.6% in these studies; estimates may vary widely because of differences in ascertaining delirium cases.^13^, ^14^, ^15^ A Danish EHR study examining delirium incidence from 1995 to 2011 reported an overall decrease in incidence rate of delirium in the whole psychiatric population, reaching 0.8 cases per 100 person‐years in 2011.^16^ To our knowledge, our study represents the largest UK primary‐care derived cohort of people with SMI to date, and is the first study to describe the incidence of delirium specifically in a population with SMI.

Within our cohort, receiving a diagnosis of in‐hospital delirium was associated with older age at study entry, SMI diagnosis of BPAD or other psychosis relative to schizophrenia, greater number of prescribed antipsychotics and more physical comorbidities. This is in line with a previous systematic review which demonstrated associations between delirium and older age, physical comorbidity burden and neuroleptic medications.^2^ Similarly, a background of bipolar disorder conferring higher odds of delirium relative to schizophrenia has been reported previously.^14^ Patients with a prior diagnosis of schizophrenia, in particular, may be at higher risk of diagnostic overshadowing in acute hospitals. Our finding that the category of ‘other psychosis’ may confer higher odds of delirium requires cautious interpretation. Whilst this may reveal differential risks between different SMI, given that the ‘other psychoses’ group makes up the majority of the subgroup diagnosed with delirium *before* an SMI, it may be that in some cases the ‘other psychosis’ diagnosis is a diagnostic and subsequent coding error in response to psychotic symptoms experienced during delirium rather than a distinct mental illness. Going forward, this highlights a need to interpret outcomes in this ‘other psychoses’ group in EHR studies with care.

A few previous studies in various settings have assessed whether a pre‐existing psychiatric disorder is a risk factor in itself for developing delirium; with some reporting an increased risk and others demonstrating no association.^22^, ^48^, ^49^, ^50^, ^51^, ^52^, ^53^, ^54^ Findings from these studies are difficult to interpret as often it is unclear how ‘psychiatric illness’ has been defined, and numbers of patients with SMI such as a psychotic disorder or bipolar disorder are often very small. One recent large community‐based case–control study examining risk factors for delirium found pre‐existing ‘serious mental illness’ to have the highest association with an odds ratio of 6.9.^22^ However, how the authors have defined ‘serious mental illness’ and whether this includes depression is unclear. Thus, whether a pre‐existing SMI as defined by QOF represents a true risk factor for delirium is yet to be established. Our cohort could be used to further examine this by comparing delirium incidence with a matched non‐SMI group.

Strengths of our study include its size, particularly as compared with previous EHR derived cohorts of people with SMI,^55^, ^56^, ^57^ and the use of both GOLD and Aurum CPRD databases. We found that whilst Aurum covers a smaller geographical area (England only), and had a lower proportion of linkage to other datasets than GOLD in this study, Aurum contributed a higher number of patients with SMI to the cohort (65%) than GOLD, and had certain advantages over GOLD such as better recorded ethnicity data, given its data is more recent (see Appendix S1). Furthermore, CPRD linkage from Aurum to other databases has improved in subsequent data releases.^58^ Thus, as increasing numbers of primary care practices move over to the EMIS system, further studies exploring delirium in particular populations can be conducted using linked Aurum‐based datasets. In addition, few previous studies have used EHRs to explore delirium occurrence,^16^, ^22^, ^23^, ^24^ thus our investigation contributes to expanding the methodology of delirium study. Using linked primary and secondary care datasets strengthens methodology further as it enables the measurement of exposure variables not captured in hospital records in order to explore relevant associations and enables the inclusion of patients who do not attend hospital during follow‐up.

There are several limitations to our study. We ascertained delirium incidence retrospectively using recorded ICD‐10 codes in HES data. Whilst this is a widely used method,^20^ it is important to note its limitations compared with prospective screening methods. As a proportion of total hospital admissions in our dataset (*n* = 286,235), delirium occurred in 0.59% (1689). This is lower than that reported in prospectively or cross‐sectionally screened inpatient samples, both of general hospital populations where occurrence varies from 11% to 42%,^59^ and psychiatric inpatients where the proportion is 1.4%–7.6%.^13^, ^16^ Interestingly, the proportion of delirium found in our sample is similar to that reported in other EHR derived retrospective cohorts; 0.6%^22^ and 0.9%,^23^ suggesting that using recorded diagnoses in EHRs significantly under‐estimates incidence. This may be because it relies on accurate diagnosis, documentation of this diagnosis and extraction of the diagnosis by clinical coders, and a degree of attrition is likely at each stage.^60^ It is interesting to note that the steep increase in incidence found in our study from 2010 onwards coincides with publication of NICE delirium guidelines and the ‘Think Delirium’ campaign,^44^; an urgent call to improve recognition of delirium due to widespread under‐recognition, poor outcomes and associated costs.^45^ Pendlebury et al recently reported an improvement in the sensitivity of hospital delirium coding from 12.8% in 2010 to 60.2% in 2018 in response to a multicomponent intervention including introducing regular screening and educational seminars.^46^ Thus, it may be that our estimates of incidence are more accurate in more recent years as awareness and screening for delirium have improved.^47^

A further limitation is that we identified delirium only using secondary‐care HES data, and not within primary‐care CPRD data. We chose secondary‐care delirium as a threshold as it captures a level of severity that warrants general or psychiatric hospital admission. However, delirium is at times diagnosed and managed in the community,^61^ thus it may be that collecting data from both primary and secondary care sources provides a more accurate measure of incidence.

Exploration of the temporal relationship between SMI and delirium diagnosis revealed a sizeable minority (18.1%) diagnosed with delirium first. Thus, it is unclear in these cases whether an undiagnosed or prodromal true SMI preceded the delirium, whether a true SMI followed the delirium or whether the SMI diagnostic code is a coding error of psychiatric symptoms experienced during delirium. Similarly, the antipsychotics variable included all antipsychotics prescribed during the follow‐up period. Therefore, it is not clear whether the association between delirium and more prescribed anti‐psychotics in this population represents a pre‐disposing factor for delirium or a consequence of delirium. Further prospective studies in this population would help to clarify the temporal direction of these relationships.

In conclusion, little is known about the relationship between SMI and acute delirium. Our study provides an initial description of the incidence and factors associated with delirium in this population using linked electronic healthcare records. Through utilising large, linked databases, our study provides a more comprehensive approach for studying patterns of delirium incidence and factors associated with this. Further prospective study is needed to accurately measure delirium occurrence in this population, to establish whether a background of SMI increases risk, and whether existing delirium diagnostic tools are appropriate for this population.

## AUTHOR CONTRIBUTIONS

Yehudit Bauernfreund formulated the research question together with Elizabeth L. Sampson and David Osborn. Graziella Favarato and Joseph F. Hayes identified the SMI cohort and compared data between GOLD and Aurum. Yehudit Bauernfreund carried out data cleaning and linkage of HES‐APC data with oversight from Naomi Launders, Elizabeth L. Sampson and David Osborn. Yehudit Bauernfreund carried out analysis with oversight from Naomi Launders, Joseph F. Hayes, Elizabeth L. Sampson and David Osborn. All authors have contributed to all iterations of the manuscript.

## CONFLICT OF INTEREST

Joseph F. Hayes has received consultancy fees from Wellcome Trust and Juli Health, other authors report no conflict of interest.

### PEER REVIEW

The peer review history for this article is available at https://publons.com/publon/10.1111/acps.13480.

## Supporting information


**Appendix S1** Supporting Information

## Data Availability

The anonymised patient‐level data used for this project cannot be shared for reasons of information governance. However, data can be obtained by application to Clinical Practice Research Datalink. Code lists are available from the corresponding author on request.
