# Supplementary material for: Incidence and associations of hospital delirium diagnoses in 85,979 people with severe mental illness: A data linkage study
Source: Acta Psychiatr Scand. 2022 Aug 5;147(5):516–26. doi: 10.1111/acps.13480 (PMC10952251; doi:10.1111/acps.13480)
Supplement: Supplementary file 1 — Appendix S1 Supporting Information [file ACPS-147-516-s001.docx]

Supplementary material

**Appendix 1** – Characteristics of whole SMI CPRD cohort and analysis for linkage bias

**SUPPLEMENTARY TABLE 1**: Characteristics of SMI participants by data source

|  |  | **SMI in CPRD GOLD (N=87,255)** | |  |  | | | |
| --- | --- | --- | --- | --- | --- | --- | --- | --- |
|  |  |  |  |  | **SMI** | **in CPRD Aurum (N=161,792)** | |  |
|  |  | n  (participants)  or median % or (IQR) | |  | n  (participants) or median | | % or (IQR) | |
| **Year of birth** |  | 1962 | (1945-1974) |  | 1964 | | (1947-1977) | |
| **Sex** | Male | 44,932 | 51.5 |  | 84,082 | | 52.0 | |
|  | Female | 42,323 | 48.5 |  | 77,710 | | 48.0 | |
| **Ethnicity** | Asian | 1,625 | 1.86 |  | 7,787 | | 4.81 | |
|  | Black | 1,875 | 2.15 |  | 10,678 | | 6.60 | |
|  | White | 34,542 | 39.6 |  | 85,137 | | 53.6 | |
|  | Mixed | 510 | 0.58 |  | 2,659 | | 2.64 | |
|  | Other | 2,117 | 2.43 |  | 3,084 | | 1.91 | |
| **Cohort entry** | Calendar year | 2001 | (2000-2008) |  | 2004 | | (2000-2011) | |
|  | At age (years) | 42 | (31 - 58) |  | 42 | | (30-57) | |
| **Cohort exit** | Calendar year | 2013 | (2008-2018) |  | 2017 | | (2010-2018) | |
|  | At age (years) | 51 | (38-67) |  | 51 | | (37-67) | |
| **Follow-up** | Duration (years) | 6.7 | (3.1-13.1) |  | 6.2 | | (2.9-13.1) | |
| **SMI diagnosis** | |  |  |  |  | |  | |
| Schizophrenia | | 28,522 | 32.7 |  | 52,046 | | 32.2 | |
| BPAD | | 27,620 | 31.7 |  | 53,343 | | 33.0 | |
| Other Psychosis | | 31,063 | 35.6 |  | 56,403 | | 34.9 | |
| **Antipsychotic prescriptions during follow-up** | |  |  |  |  | |  | |
| None | | 26,798 | 30.7 |  | 52,456 | | 32.4 | |
| 1-2 Oral | | 42,860 | 49.1 |  | 80,350 | | 49.7 | |
| >2Oral +/- depot | | 17,597 | 20.2 |  | 28,986 | | 17.9 | |
| **Death during follow-up** | | 13,095 | 15.0 |  | 22,592 | | 14.0 | |
| At age (years) | | 74 | (61 – 84) |  | 75 | | (62 – 84) | |
| Year of death | | 2009 | (2005 – 2013) |  | 2011 | | (2006 – 2015) | |

**Supplementary table 1 footnote:** *CPRD, Clinical Practice Research Datalink; SMI, Severe Mental Illness; CI, confidence interval; IQR, interquartile range; BPAD, Bipolar Affective Disorder; FU, follow-up.*

**SUPPLEMENTARY FIGURE 1**: number of SMI patients and GP practices (on top of bar) by region and data source.


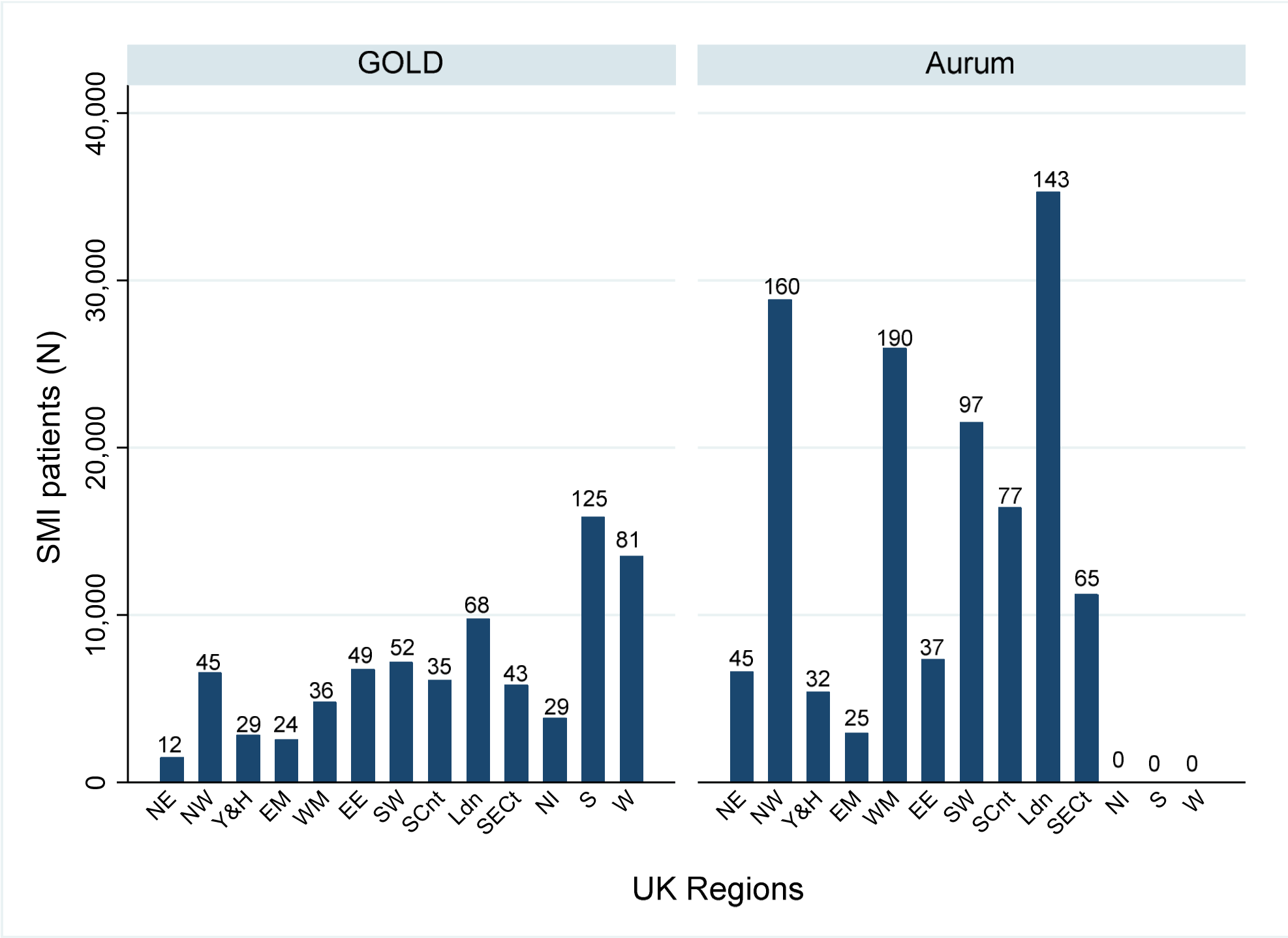


**Supplementary figure 1 footnote:** *NE, North East; NW, North West; Y&H, Yorkshire & the Humber; EM, East Midlands; WM, West Midlands; EE , East of England; SW , South West; SW , South West; SCnt , South Central; Ldn, London; SECt , SouthEast Coast; NI , Northern Ireland; S, Scotland; S, Scotland; W, Wales.*

**SUPPLEMENTARY TABLE 2:** Ethnicity breakdown within our dataset by source

|  | **GOLD** | **Aurum** | **Total** |
| --- | --- | --- | --- |
| Asian | 1,625 (1.86) | 7,787 (4.81) | 9,412 (3.78) |
| Black | 1,875 (2.15) | 10,678 (6.60) | 12,553 (5.04) |
| Mixed | 510 (0.58) | 2,659 (1.64) | 3,169 (1.27) |
| Other | 2,117 (2.43) | 3,084 (1.91) | 5,201 (2.09) |
| White | 34,542 (39.6) | 85,137 (52.6) | 119,679 (48.1) |
| Missing | 46,586 (53.4) | 52,447 (32.4) | 99,033 (39.8) |
| **Total** | **87,255** | **161,792** | **249,047** |

**SUPPLEMENTARY FIGURE 2:** Percentage of records with complete ethnicity recording by year of end of follow – up

**SUPPLEMENTARY TABLE 3:** Comparison of those eligible for HES linkage to those not eligible for HES linkage.

|  | **Eligible for linkage** | | **Not eligible for linkage** | |
| --- | --- | --- | --- | --- |
| **N** | 85,979 | | 163,068 | |
|  | N/median | % (95% CI) / IQR | N/median | % (95% CI) / IQR |
| **Source**  GOLD  Aurum | 40,738  45,241 | 47.4 (47.0 – 47.4)  52.6 (52.3 – 53.0) | 46,517  116,551 | 28.5 (28.3 – 28.7)  71.5 (71.3 – 71.7) |
| **Age at study entry (years)** | 42 | 30 - 58 | 42 | 31 - 57 |
| **Follow-up time** | 6.2 | 2.9 – 12.4 | 6.5 | 3.0 – 13.6 |
| **Gender**  Female  Male | 41,693  44,286 | 48.5 (48.2 – 48.8)  51.5 (51.2 – 51.8) | 78,340  84,728 | 48.0 (47.8 – 48.3)  52.0 (51.7 – 52.2) |
| **Ethnicity**  Asian  Black  White  Mixed  Other | 3,870  4,676  74,358  1,088  1,987 | 4.5 (4.4 – 4.6)  5.4 (5.3 – 5.6)  86.5 (86.3 – 86.7)  1.3 (1.2 – 1.3)  2.3 (2.2 – 2.4) | 5,543  7,877  144,358  2,081  3,214 | 3.4 (3.3 – 3.5)  4.8 (4.7 – 4.9)  88.5 (88.4 – 88.7)  1.3 (1.2 – 1.3)  2.0 (1.9 – 2.0) |
| **SMI diagnosis**  BPAD  Schizophrenia  Other psychosis | 27,848  27,570  30,561 | 32.4 (32.1 – 32.7)  32.1 (31.8 – 32.4)  35.5 (35.2 – 35.9) | 53,165  52,998  56,905 | 32.6 (32.4 – 32.8)  32.5 (32.3 – 32.7)  34.9 (34.7 – 35.1) |
| **No. of antipsychotics**  None  1-2  >2 +/- depot | 28,111  42,456  15,412 | 32.7 (32.4 – 33.0)  49.4 (49.0 – 49.7)  17.9 (17.7 – 18.2) | 51,143  80,754  31,171 | 31.4 (31.1 – 31.6)  49.5 (49.3 – 49.8)  19.1 (18.9 – 19.3) |
| **≥1 comorbidity on Charlson Comorbidity Index** | 25,769 | 30.0 (29.7 – 30.3) | 48,525 | 30.0 (29.5 – 30.0) |
| **Died during FU** | 11,917 | 13.9 (13.6 – 14.1) | 23,770 | 14.6 (14.4 – 14.7) |
| **Age at death (years)** | 76 | 62 – 85 | 75 | 61 – 84 |

**Supplementary table 3 footnote:** 95% confidence intervals displayed around proportions*. SMI, Severe Mental Illness; CI, confidence interval; IQR, interquartile range; BPAD, Bipolar Affective Disorder; FU, follow-up; HES, Hospital Episodes Statistics*

**Appendix 2 –** Systematic cleaning of HES-APC data to identify unique diagnostic episodes

**SUPPLEMENTARY FIGURE 3**: Flowchart demonstrating systematic cleaning of HES-APC data for linkage.

Diagnostic episodes* in HES-APC linked to subset eligible for linkage

**3,387,566**

Diagnostic episodes excluded:

18,359 occurred when patient <16 years

144,489 prior to start of follow – up

1,643,970 occurred outside of active CPRD time**

73,333 classified as treatment over series of admissions or maternity delivery admissions

28,068 duplicates (i.e. same unique patient identifier, unique admission identifier, unique episode identifier, episode start and end dates and ICD diagnostic code, or same in all categories other than unique episode identifier )

42 error end date before start date

3210 excluded as duplicate CPRD IDs to one HES ID

**1,911,471**

Diagnostic episodes in HES-APC after data cleaning

**1,476,095**

Diagnostic episodes not delirium related

**1,473,199**

**2,896** unique delirium diagnostic episodes

(2,889 unique delirium episodes,

1,689 unique delirium related admissions,

1,337 unique patients with ≥1 delirium admission during follow-up)

**Supplementary figure 3 footnote:** *HES-APC, Hospital Episodes Statistics Admitted Patient Care; CPRD, Clinical Practice Research Datalink; ID, identification number*

*Within HES-APC data, each hospitalisation can consist of one or more episodes. For each episode, up to 20 diagnoses may be recorded. ”Diagnostic episode” refers to individual diagnoses within individual episodes.

**We excluded episodes recorded in HES that occurred outside of the time a patient was registered within a CPRD practice as we were not able to account for demographic or clinical variables during this period for the purposes of analysis.

**Appendix 3** – ICD-10 codes used to identify delirium and yearly incidence rates

**SUPPLEMENTARY TABLE 4:** ICD 10 codes used to identify delirium in HES-APC data with numbers of each code identified in our cohort displayed

| **ICD-10 code** | **Number of diagnostic episodes identified** |
| --- | --- |
| **F05 Delirium, not induced by alcohol and other psychoactive substances** |  |
| F05.0 Delirium, not superimposed on dementia, so described | 168 |
| F05.1 Delirium, superimposed on dementia | 311 |
| F05.8 Other delirium | 152 |
| F05.9 Delirium, unspecified | 2059 |
| **G Acute encephalopathy** |  |
| G04.30 Acute necrotising hemorrhagic encephalopathy | 0 |
| G04.31 Post infectious acute necrotising hemorrhagic encephalopathy | 0 |
| G04.32 Post immunization acute necrotising hemorrhagic encephalopathy | 0 |
| G92 Toxic encephalopathy | 20 |
| G93.4 Acute encephalopathy, unspecified | 186 |
| G93.41 Metabolic encephalopathy | 0 |
| G93.49 Other encephalopathy | 0 |
| **Total:** | 2,896* |

**Supplementary Table 4 footnote:** *ICD, International Classification of Diseases*

*7 episodes had both an F05 and G diagnostic code

**SUPPLEMENTARY TABLE 5:** Yearly incidence rates (cases per 100 person-years for each year of follow-up) of delirium within our linked cohort, with 95% confidence intervals displayed

| **Year** | **No. of delirium admissions** | **Participants in cohort that year** | **Person-years within that year of cohort** | **Delirium admissions per 100 person – years (95% CI)** |
| --- | --- | --- | --- | --- |
| 2000 | 13 | 34,821 | 33,538.82 | 0.04 (0.02 – 0.07) |
| 2001 | 31 | 37,572 | 35,177.62 | 0.09 (0.06 – 0.13) |
| 2002 | 33 | 38,901 | 36,236.22 | 0.09 (0.06 – 0.13) |
| 2003 | 28 | 39,987 | 37,248.42 | 0.08 (0.05 – 0.11) |
| 2004 | 33 | 40,910 | 38,032.27 | 0.09 (0.06 – 0.12) |
| 2005 | 34 | 41,406 | 38,457.64 | 0.09 (0.06 – 0.12) |
| 2006 | 25 | 42,089 | 38,814.89 | 0.06 (0.04 – 0.10) |
| 2007 | 46 | 42,382 | 39,203.7 | 0.12 (0.09 – 0.16) |
| 2008 | 49 | 42,732 | 39,420.48 | 0.12 (0.09 – 0.16) |
| 2009 | 64 | 42,636 | 39,401.46 | 0.16 (0.13 – 0.21) |
| 2010 | 58 | 42,700 | 39,169.18 | 0.15 (0.11 – 0.19) |
| 2011 | 86 | 42,332 | 38,449.81 | 0.22 (0.18 – 0.28) |
| 2012 | 112 | 41,445 | 38,065.13 | 0.29 (0.24 – 0.35) |
| 2013 | 159 | 40,391 | 36,220.48 | 0.44 (0.37 – 0.51) |
| 2014 | 183 | 37,732 | 33,982.03 | 0.54 (0.46 – 0.62) |
| 2015 | 211 | 35,669 | 32,148.8 | 0.66 (0.57 – 0.75) |
| 2016 | 226 | 33,616 | 30,006.27 | 0.75 (0.66 – 0.86) |
| 2017 | 298 | 31,262 | 28,479.17 | 1.05 (0.93 – 1.17) |

**Supplementary Table 5 footnote:** *CI, confidence interval*

**Appendix 4** – Sensitivity analysis

**SUPPLEMENTARY TABLE 6:** Odds ratios for receiving an in-hospital delirium code within group who had any hospital admission during follow-up (n=57,354).

| **Exposure** | **Unadjusted** | | | **Age and sex adjusted** | | | **Fully adjusted** | | |
| --- | --- | --- | --- | --- | --- | --- | --- | --- | --- |
|  | **Odds ratio** | **95% CI** | **p-value** | **Odds ratio** | **95% CI** | **p-value** | **Odds ratio** | **95% CI** | **p-value** |
| Age at study entry | 1.05 | 1.05 – 1.05 | <0.001 | 1.05 | 1.04 – 1.05 | <0.001 | 1.05 | 1.05 – 1.05 | <0.001 |
| Gender  - Male  - Female | ref  1,53 | 1.37 – 1.71 | <0.001 | ref  1.03 | 0.92 – 1.16 | 0.618 | ref  0.97 | 0.86 – 1.09 | 0.609 |
| Ethnicity   - White - Asian - Black - Mixed - Other | 1  0.64  0.61  0.56  1.20 | 0.46 – 0.90  0.45 – 0.84  0.29 – 1.07  0.85 – 1.70 | 0.010  0.002  0.081  0.300 | 1  1.03  1.06  1.11  1.32 | 0.73 – 1.44  0.77 – 1.46  0.57 – 2.16  0.93 – 1.88 | 0.871  0.714  0.763  0.117 | 1  1.04  1.13  1.17  1.32 | 0.74 – 1.46  0.82 – 1.56  0.60 – 2.29  0.93 – 1.89 | 0.817  0.452  0.639  0.119 |
| SMI diagnosis   - Schizo-phrenia - BPAD - Other Psychosis | ref  1.40  1.60 | 1.21 – 1.62  1.39 – 1.83 | <0.001  <0.001 | ref  1.41  1.40 | 1.21 – 1.63  1.21 – 1.61 | <0.001  <0.001 | ref  1.52  1.47 | 1.31 – 1.76  1.27 -1.70 | <0.001  <0.001 |
| Antipsychotics during follow-up   - None - 1-2 APs - >2 APs +/- depot | ref  1.28  1.42 | 1.12 – 1.47  1.21 – 1.67 | <0.001  <0.001 | ref  1.38  1.72 | 1.20 – 1.59  1.47 – 2.02 | <0.001  <0.001 | ref  1.40  1.84 | 1.21 – 1.60  1.57 – 2.17 | <0.001  <0.001 |
| Physical comorbidities (Charlson Index) | 1.50 | 1.42 – 1.58 | <0.001 | 1.07 | 1.01 – 1.14 | 0.022 | * |  |  |

*not included in multivariate logistic regression as felt to lie on causal pathway

**Supplementary Table 6 footnote:** *OR, odds ratio, CI, confidence interval; BPAD, Bipolar Affective Disorder; APs, antipsychotics*
